# Cure of Sore Nipples

**Published:** 1842-12-03

**Authors:** 


*Cure of Sore Nipples.*—Mr. J. H. Horne informs us that he had anticipated, in some printed remarks, "the injury which so valuable a remedy for sore nipples as the tincture of catechu must inevitably receive by using it injudiciously." Mr. Horne adds, "Mr. Davis's cases, published in the 'Students' Number' of the Lancet, Oct. 1st, are proof. I wrote,—the tincture of catechu has lately been introduced unconditionally as a specific for sore nipples. As such, disappointment must frequently be the consequence. Should it be prescribed in the inflammatory or early stage, it will increase the sufferings of the patient rather than ameliorate them, for the nipple becomes more hot, dry, and hard, and fresh cracks quickly follow, not a little heightened by the state of the system. After the state of excitement is gone by, and the nipple assumes a sloughy condition, then is the time when the tincture of catechu exerts its beneficial influence. The following linimtne (both very old remedies for sore nipples) should precede the use of it, viz.:—

℞ Sub-borat. of soda, grs. x.;

Hot water, ℥i;

Oil of almonds, ℥vij.

Mix well, and make a liniment.

"I know not to whom we are indebted for these two very excellent remedies, but this much I know, that in my own practice, I can conscientiously state, that I never prescribed either without the patient's deriving immediate relief, and very shortly afterwards a cure; and if the practitioner do his duty they will be found to work theirs most assuredly. The directions that I enforce are these:—With a warm and moist sponge cleanse the nipple before the infant sucks, and also immediately after sucking, and then, with a camel-hair brush, apply the application; and on no condition do I allow the breasts to be neglected, or take for granted, from the nurse, that all is right, without seeing with my own eyes, and judging for myself.—*London Lancet*, Oct. 15, 1842.

